# Quantitative Investigation of Nitrosamine Drug Substance‐Related Impurities (NDSRIs) Under Artificial Gastric Conditions by Liquid Chromatography–Tandem Mass Spectrometry and Structure–Activity Relationship Analysis

**DOI:** 10.1002/dta.3874

**Published:** 2025-03-12

**Authors:** Matthias Vogel, Sylvia E. Escher, Emanuel Weiler, Anke Londenberg, Uwe Deppenmeier, Rhys Whomsley

**Affiliations:** ^1^ Federal Institute for Drugs and Medical Devices Bonn Germany; ^2^ Fraunhofer Institute for Toxicology and Experimental Medicine Hannover Germany; ^3^ Institute of Microbiology and Biotechnology University of Bonn Bonn Germany; ^4^ European Medicines Agency Amsterdam The Netherlands

**Keywords:** analysis, liquid chromatography, mass spectrometry, NDSRIs, nitrosamines, validation

## Abstract

The presence of nitrosamines in numerous human medicinal products (HMP) has recently emerged as a cause for concern. Following the initial discovery of carcinogenic low molecular weight nitrosamines such as nitrosodimethylamine (NDMA) in HMP, regulatory authorities worldwide requested marketing authorization holders to perform risk assessments for the presence of nitrosamines in their products. The initially observed contaminations—mainly low molecular weight nitrosamines—were generated by organic solvent impurities or by‐products from synthesis and nitrite carried over to finished products (FP). More recently, complex nitrosamine drug substance‐related impurities (NDSRIs) have been reported arising from direct nitrosation of active pharmaceutical ingredients (APIs) at secondary amine groups in the presence of nitrite derived from excipients. In addition, an alternative route of API nitrosation is conceivable due to interaction with gastric acid and physiological nitrite after drug intake. Within this study, 13 secondary amine bearing APIs were selected to individually identify susceptibilities for nitrosation by using high physiological limit values in terms of pH and nitrite. Therefore, artificial gastric media were fortified with 200 μM sodium nitrite and increasing concentrations of APIs at pH 3.15 and 37°C for 2 h. All NDSRI concentrations were quantitatively determined via validated liquid chromatography–tandem mass spectrometry (LC–MS/MS) methodology. Additionally, time‐dependent nitrosations of selected APIs were monitored to kinetically assess the proportion of NDSRIs after the gastric passage. All results and observations were further processed by means of structure activity relationship (SAR) predictions to identify highly susceptible compounds in the group of concern.

## Graphical abstract

The study provides information about the impact of physiological gastric conditions on the unbeneficial occurrence of nitrosamine drug substance–related impurities (NDSRIs). Both main factors for nitrosation in the form of acidic conditions and the presence of nitrite were adequately chosen for triggering the conversion of drug compounds into NDSRIs. All steps were qualitatively and quantitatively monitored by means of liquid chromatography–tandem mass spectrometry.

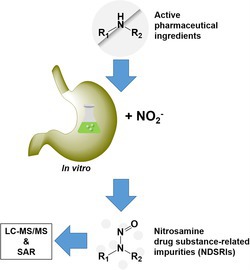

## 1 Introduction

**FIGURE 1:**
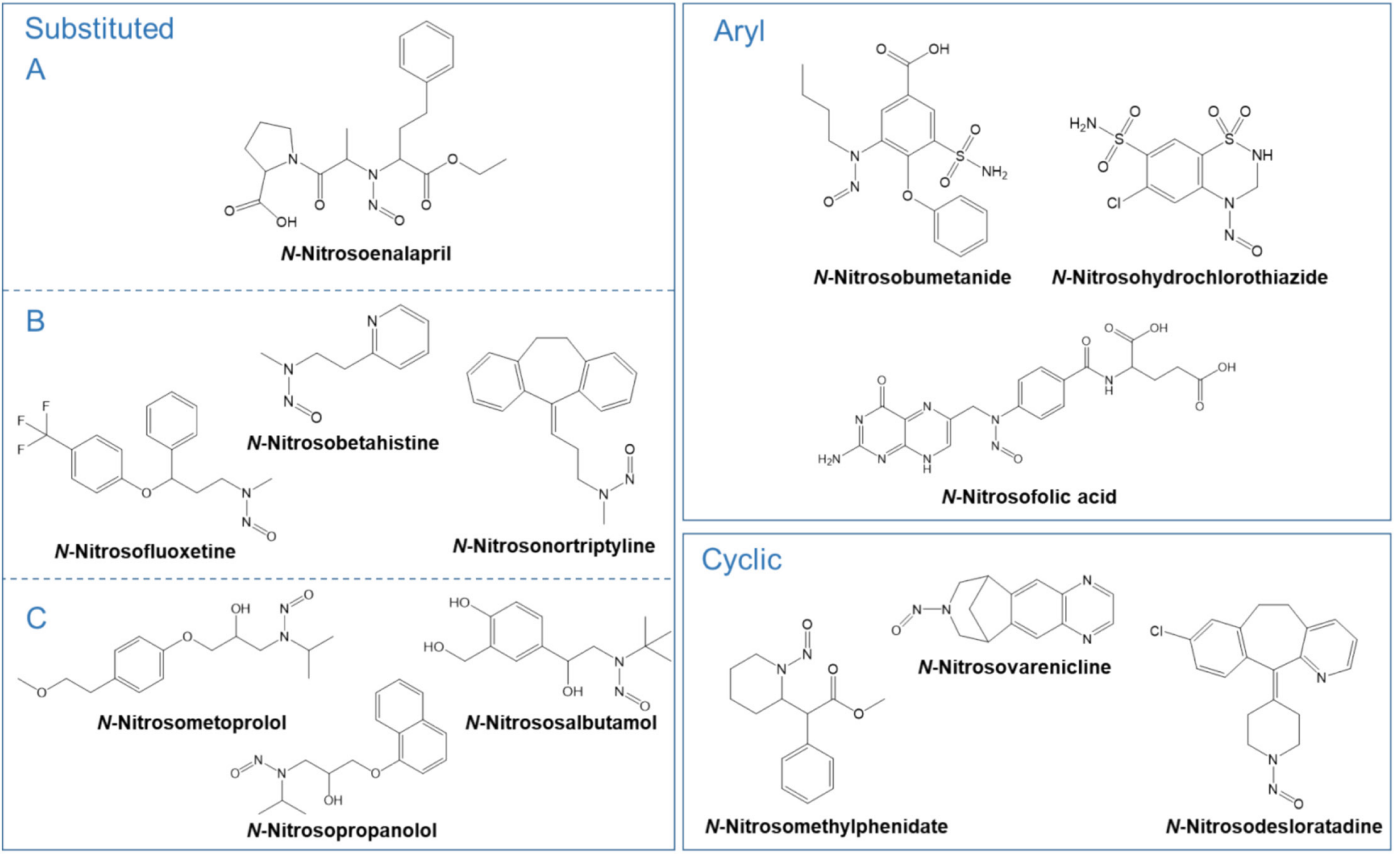
Overview of investigated NDSRIs with aryl, cyclic embedded, or aliphatically substituted residues ([A] R_1_ and R_2_ are both complex substituted; [B] R_1_ = methyl, R_2_ = complex aliphatically substituted; [C] R_1_ = isopropyl/tert‐butyl, R_2_ = complex aliphatically substituted) adjacent to the nitrosoamine group.

In the last decades, the modification of substances within consumables due to nitroso group (NO) introduction had a significant impact on various toxicological classifications [1, 2, 3]. Nitrosation reactions, in particular at secondary amine groups, can significantly influence the mutagenic characteristics and potential risks associated with these affected compounds, generally termed as “nitrosamines.” Since the first detection of the mutagenic nitrosamine nitrosodimethylamine (NDMA) in medicinal products in 2018, the awareness of health authorities to nitrosamines has led to far‐reaching regulatory measures to prevent these contaminations [4]. The severity of these contaminations progressively increased from low‐molecular weight nitrosamines (e.g., NDMA, NDEA) to nitrite affected derivatization reagents (e.g., nitrosomethylpiperazine in rifampicin) and finally resulted in the detection of directly nitrosated pharmaceuticals containing secondary amine groups (e.g., nitrosovarenicline in Champix®). To date, the number of so‐called nitrosamine drug substance‐related impurities (NDSRIs) reported to the European Medicines Agency is continuously growing. In silico analyses have suggested that 40.4% out of 12,000 investigated drugs can theoretically form NDSRIs [5]. In contrast to the wealth of scientific data for smaller nitrosamines, little to no information is available for the novel class of NDSRIs. In particular, toxicological evidence in the form of mutagenicity assessments, physiochemical evaluations for API vulnerabilities to nitrosation, and valid analytical methods for compound detection will be of utmost importance in the future [6]. For these purposes, it is necessary to implement predictive models in advance, where combinations of structure–activity relationship (SAR) investigations and confirmative LC–MS/MS play a major role. In general, one of the most decisive factors for *N*‐nitrosation enhancement or suppression, which were amply investigated, is the nucleophilicity of the NH‐group [7]. Thereby, enhanced reactivity is often accompanied with low pk_a_ values or low basicity, for example, aromatic secondary amines. Intramolecular factors such as adjacent electron donating or withdrawing effects (i.e., positive and negative inductive effects), mesomeric stabilization, and potential hydrogen bonding interactions play a critical role in modulating the reaction kinetics [6]. Additionally, the usage of API salt forms (e.g., hydrochlorides) accompanied by respective protonation grades of the secondary amines or conditions and excipients during drug formulation can diminish nitrosation rates [8]. The identification of structural inhibitory and protective effects can be advantageous in maintaining product stability, mitigating the risk of undesirable N‐nitrosamine formation, and ensuring pharmaceutical product safety and quality. Addressing the issue of nitrosamine contamination requires a multifaceted approach combining root cause analysis and rigorous quality control measures. Even when pharmaceutical products are devoid of nitrosamines during production, some compounds may undergo nitrosation in the acidic environment of the stomach [9]. This phenomenon has sparked investigations into the potential sources of nitrosating agents in the human digestive system [10, 11, 12, 13]. The interaction between pharmaceutical ingredients and gastric fluids could lead to nitrosamine formation, highlighting the need for a deeper understanding of these chemical reactions. This study provides valuable insights in the context of highly sensitive NDSRI detection via modern LC–MS/MS technologies as well as first knowledge about API to be prone to nitrosation. In this study, 13 APIs with varying secondary amine residues were investigated, following analytical method implementation and validation, by incubating equal API concentrations under physiological conditions at pH 3.15, 200 μM nitrite, and 37°C in artificial gastric juice. The pivotal consideration guiding API selection was to maximize the inclusion of diverse secondary amine groups, ensuring a high degree of heterogeneity. Hence, secondary amines spanning aliphatic, cyclic, and aromatic variants were incorporated (Figure 1). In addition to LC–MS/MS determinations, the acquired conversion rates underwent in silico SAR analysis, unveiling discernible trends in API nitrosation. The findings from this study are expected to have valuable implications for potential analytical applications and offer guidance for regulatory stakeholders and pharmaceutical manufacturers.

## 2 Experimental Section

### 2.1 Material and Chemicals

The API amitriptyline hydrochloride, enalapril maleate, betahistine hydrochloride, fluoxetine hydrochloride, hydrochlorothiazide, loratadine, metoprolol tartrate, propranolol hydrochloride, salbutamol hemisulfate, and folic acid were purchased from TCI Chemicals (Zwijndrecht, Belgium). Bumetanide and varenicline tartrate were obtained from biomol (Hamburg, Germany). Methylphenidate hydrochloride was obtained from Merck (Darmstadt, Germany) after authorization by the German Federal Opium Agency. The stable isotopically labelled APIs for the synthesis of internal standards (ISTDs) were either procured from biomol or biozol (Eching, Germany). The NDSRI reference compounds in form of nitrosonortriptyline (NNor), nitrosohydrochlorothiazide (NHCT), nitrosoenalapril (NEna), and nitrososalbutamol (NSal) were custom synthesized by Clearsynth (Mumbai, India). Nitrosofolic acid (NFolAc) were custom synthesized by Selvita (Krakow, Poland). Most NDSRIs, particularly nitrosopropranolol (NProp), nitrosometoprolol (NMeto), nitrosomethylphenidate (NMePh), nitrosobetahistine (NBeta), nitrosobumetanide (NBume), nitrosoloratadine (NLor), and nitrosofluoxetine (NFluo), were obtained from Enamine (Kyiv, Ukraine). Nitrosovarenicline (NVar) was ordered from Toronto Research Chemicals (Toronto, Canada). All reagents for the ISTD syntheses and LC–MS/MS measurements were purchased from Merck. Compounds to prepare artificial gastric juice were ordered from Carl Roth GmbH (Karlsruhe, Germany). Human HepG2 cell lines were also purchased from Merck (Darmstadt, Germany). Bacteria lysates were provided by the Institute of Microbiology and Biotechnology (University of Bonn, Bonn, Germany). Placebo tablets were ordered from Fagron GmbH from a local pharmacy.

### 2.2 Stock Solutions and Synthesis of Internal Standards

All APIs as well as NDSRIs were dissolved in dimethylsulfoxide (DMSO) with 5 mM stock solutions. For ISTD syntheses, 5 μL of each stable isotopically labelled API (10 mg/mL DMSO) were fortified with 2 mg of sodium nitrite and 5 μL of concentrated hydrochloric acid in an Eppendorf tube. The mixture was filled up with double distilled water to 100 μL and slowly shaken at 50°C for 2 h. After reaction, the mixture was made up to 1 mL with bidoublet distilled water and purified via 1 mL Chromabond® C18 (Macherey‐Nagel, Dueren, Germany) solid phase extraction columns. The acetonitrile eluents of all ISTDs were pooled for further processing and stored at −20°C. All synthesis steps were performed under safety conditions in the fume hood. Identities were verified by comparison with reference spectra or structure eluciations via occurrences of specific product ions.

### 2.3 Artificial Gastric Juice Incubations and Sample Work‐Up

Artificial gastric juice were prepared and modified in reference to SHIME reactor medium from Molly et al. and van de Wiele et al. [14, 15]. One gram mucin, 2.5 g sodium bicarbonate, 2 g sodium chloride, 5.6 g dipotassium hydrogenphosphate, 4.4 g potassium dihydrogenphopshate, 1 mL salt solution (8 g calcium chloride; 8 g magnesium sulfate heptahydrate ad 1000 mL), 1 g ammonium chloride, and 50 mg pepsin (added after autoclaving) were made up to 1000 mL with double distilled water and subsequently autoclaved. pH and amounts of nitrite were adjusted with hydrochloric acid and sodium nitrite successively prior to the experiments. After fortifying respective concentrations of APIs into 1 mL artificial gastric juice (pH 3.15, 200 μM nitrite) and incubating for 2 h, reactions were stopped by adding 2 μL sulfamic acid. Technical replicates were further processed by solid phase extraction (SPE) using Chromabond® HLB 96‐well plates (Macherey‐Nagel, Dueren, Germany) following the manufacturer's instructions. Acetonitrile eluents were collected in 2 mL 96 deep well plates and evaporated with nitrogen _(g)_ at 55°C to dryness. All samples were reconstituted in 100 μL 5 mM ammonium acetate/0.1% acetic acid.

### 2.4 Liquid Chromatography–Tandem Mass Spectrometry

LC–MS/MS measurements were performed with a Sciex QTRAP® 6500 triple quadrupole MS (Darmstadt, Germany) coupled to a Shimadzu Nexera® UPLC (Duesseldorf, Germany). For chromatographic separation, an Agilent Poroshell® C8 (50 × 3 mm, 2.7 μm) was utilized and equipped with a 4 × 3‐mm Universal RP Guard Column (Macherey‐Nagel, Dueren,). The eluents consisted of (A) 5 mM ammonium acetate/0.1% acetic acid buffer (pH 3.5) and (B) acetonitrile. Twelve‐minute gradient elution was performed with *T*_min_/B% 0.2/1; 10/100; 10.01/1; 12/1 with a flow rate of 0.45 mL/min. All multiple reaction monitoring experiments (MRM) were performed under positive and negative electrospray ionization at 500°C and needle voltage of 5500 and −4500 eV, respectively. Declustering potentials (DP) were set to 40 eV. An overview of all monitored quantifier and qualifier ion transitions is listed in Table 1.

**TABLE 1:** Overview of final MRM ion transitions for all NDSRI analytes with respective declustering potentials, collision energies, and ionization modes. Red marked *m/z* values indicate the neutral losses of nitroso groups or HNO.

ID (NDSRIs + ISTDs)	*m/z* [M + H]^+^	*m/z* [M‐H]^−^	*m/z* [M + Na]^+^	*m/z* Quant	*m/z* Qual 1	*m/z* Qual 2	*m/z* Qual 3	DP (eV)	ce (eV)	Ion source	cis‐trans isomers
Nitrosofluoxetine	339.1	−	−	117.1	177.2	146.1	309.1	40	15/15/15/5	(+)ESI	−
Nitrosometoprolol	297.2	−	319.2	72.1	266.2	145.1	206.1		18/18/20/18	(+)ESI	+
Nitrososalbutamol	−	267.1	−	151.2	219.1	162.1	204.2		−22	(−)ESI	−
Nitrosovarenicline	241.1	−	−	211.1	194.1	169.1	−		20	(+)ESI	−
Nitrosohydrochlorothiazide	−	324.9	−	281.1	294	217	−		−10	(−)ESI	−
Nitrosopropranolol	289.1	−	−	145.1	259.2	215.2	128.2		5	(+)ESI	+
Nitrosomethylphenidate	263.1	−	−	84.1	232.1	129.1	−		15/5/10	(+)ESI	−
Nitrosobumetanide	−	392.1	−	318.1	362.1	270.2	−		10/10/20	(−)ESI	−
Nitrosonortriptyline	293.1	−	−	233.1	191.1	155	263.1		5/15/5/5	(+)ESI	+
Nitrosofolic acid	−	469.1	−	439.1	395	421	−		−18	(−)ESI	−
Nitrosodesloratadine	340.1	−	−	310.1	281	293	−		20	(+)ESI	−
Nitrosoenalapril	406.1	−	428.1	116.1	234.1	375.1	−		13	(+)ESI	+
Nitrosobetahistine	166.1	−	−	93.0	136.1	135.1	106.1		10/5	(+)ESI	−
d5‐Nitrosofluoxetine	344.2	−	−	182.2	122.2	−	−	40	15/15/15/5	(+)ESI	−
d7‐Nitrosometoprolol	304.2	−	326.2	79.1	273.2	152.1	−		18/18/20/18	(+)ESI	+
d9‐Nitrososalbutamol	−	276.2	−	151.1	228.1	210.4	163.1		−22	(−)ESI	−
d4‐Nitrosovarenicline	245.1	−	−	215.2	197	−	−		20	(+)ESI	−
13C,d2‐Nitrosohydrochlorothiazide	−	328.0	−	284.1	220	296	−		−10	(−)ESI	−
d7‐Nitrosopropranolol	296.1	−	−	266.2	145.1	222.2	−		5	(+)ESI	+
d3‐Nitrosomethylphenidate	266.1	−	−	84.1	235.2	129.1	−		15/5/10	(+)ESI	−
d5‐Nitrosobumetanide	−	397.1	−	323.2	367.2	−	−		10/10/20	(−)ESI	−
d3‐Nitrosonortriptyline	296.2	−	−	233.2	266.2	191.1	−		5/15/5/5	(+)ESI	+
d4‐Nitrosofolic acid	−	473.1	−	443.1	399	425	−		−18	(−)ESI	−
d4‐Nitrosodesloratadine	344.1	−	−	314.2	283.1	268.1	−		20	(+)ESI	−
d5‐Nitrosoenalapril	411.2	−	433.2	116.1	239.1	380.1	296.2		13	(+)ESI	+
d3‐Nitrosobetahistine	169.1	−	−	93.0	94.0	139.1	−		10/5	(+)ESI	−

### 2.5 Validation

Analytical validation in accordance to ICH Q2(R1) was mandatory for accurate and unambiguous determination of NDSRIs as there was no information available for validated and implemented methods for these substances [16].

#### 2.5.1 Specificity/Selectivity

NDSRI free matrices (*n* = 10) in form of pure artificial gastric juice, tap water, phosphate buffered saline (PBS, pH 7.4), 500 mg placebo tablet, organic solvents (DMSO, ACN, MeOH), pooled HepG2 cell lysates, bacteria lysates, and coffee were chosen for probing. Furthermore, all 10 samples (*n* = 10 + 10) were fortified with 1 nmol/mL of each NDSRI and the ISTD mixture to unambiguously confirm identification capability by unit resolution.

#### 2.5.2 Linearity/Range

For each NDSRI, eight concentration levels with ISTD mix (0.005, 0.025, 0.1, 0.25, 0.5, 1, 2.5, and 5 nmol/mL) were determined in artificial gastric juice (pH 3.15, 200 μM nitrite) after sample workups. Respective calibration curves and coefficients of correlations were derived to assess the method's linearity.

#### 2.5.3 LOD/LOQ

According to the ICH Q2 (R2) guideline, LOD and LOQ for each NDSRI were determined via signal‐to‐noise ratios of at least 3:1 for LOD and 10:1 for LOQ, respectively. All samples at respective concentration levels were repeated six times.

#### 2.5.4 Precision (Repeatability and Intermediate Precision)

To test the precision of the method, repeatability as well as intermediate precision were performed. The repeatability parameter was determined for each NDSRI at 0.025, 0.25, and 2.5 nmol/mL with *n* = 6 + 6 + 6. Taking repeatability into account, the intermediate precision is determined on three consecutive days and the relative standard deviations of the peak‐to‐area ratios between days 1 and 2, 1 and 3, and correspondingly 2 and 3 were calculated. The intended imprecision limits, expressed as relative standard deviations (rel SD), were set to ±15% for all concentration levels.

#### 2.5.5 Accuracy

Accuracy was calculated from the precision samples (*n* = 6 + 6 + 6, day 1) by applying the means of each concentration level to the linearity calibration curves. All results were summarized as imprecisions in percent (rel SD [%]).

#### 2.5.6 Robustness

The robustness of the method was proofed in the course of the study measurements. The initially utilized Thermo Fisher Accucore® C8 chromatographic column was replaced by an Agilent Poroshell® C8 by maintaining all LC–MS settings.

#### 2.5.7 Stability

All stock solutions were separately stored in DMSO and double distilled water at 4 and −20°C for at least 4 days to subsequently determine the intact NDSRI. Relative signal intensity reductions of each NDSRI were monitored to evaluate stability of each NDSRI.

### 2.6 Quantitative NDSRI Determination Under Artificial Gastric Conditions

The applicability of the method was tested by analyzing NDSRI emergences from selected APIs, with exception of betahistine, after nitrite treatment under optimal acidic conditions. For these purposes, concentrations of 25, 50, 100, and 200 μM of APIs were incubated in the presence of 200 μL artificial gastric juice (pH 3.15, 200 μM) at 37°C for 2 h. The reasons for selecting these incubation conditions are explained in more detail in the Results and Discussion sections. During incubation approaches, all samples were protected from light and mixed on an Eppendorf Thermomixer® (Hamburg, Germany) at 500 rpm. Reactions were stopped in accordance to the work up procedure by the addition of 2 μL sulfamic acid to perform SPE of NDSRIs. For quantification, the working range from linearity was increased to 50 nmol/mL, as the expected concentration responses from NDSRI or signal intensities were unknown. For the experiment with identical API concentrations (each at 100 μM) but exponentially increasing nitrite concentrations, incubation was likewise carried out at pH 3.15 and 37°C for 2 h. The reaction was then quenched and processed as described. For the linear regression calculations, Python version 3.9.12 was used along with the packages seaborn 0.11.2 and scipy 1.7.3.

### 2.7 In Silico Analysis of Structure Activity Relationship

#### 2.7.1 Molecular Properties of the APIs

Molecular properties were computed from the SMILES (Simplified Molecular‐Input Line‐Entry System) codes of the 12 APIs. Canonical SMILES codes were extracted from PubCHEM and controlled manually for correctness [17]. The MACCS molecular fingerprints were as implemented in the RDKit fingerprint module integrated in a KNIME workflow [18]. Several algorithms can be used to calculate compound similarity. In this case, the Tanimoto and DICE algorithms were applied [19]. All pk_a_ values for the secondary amine substructure as well as its predicted protonation state at pH 3 were calculated for all secondary amines of the tested APIs using ACD/Percepta 14.3.0.

## 3 Results and Discussion

### 3.1 Liquid Chromatography–Tandem Mass Spectrometry

**FIGURE 2:**
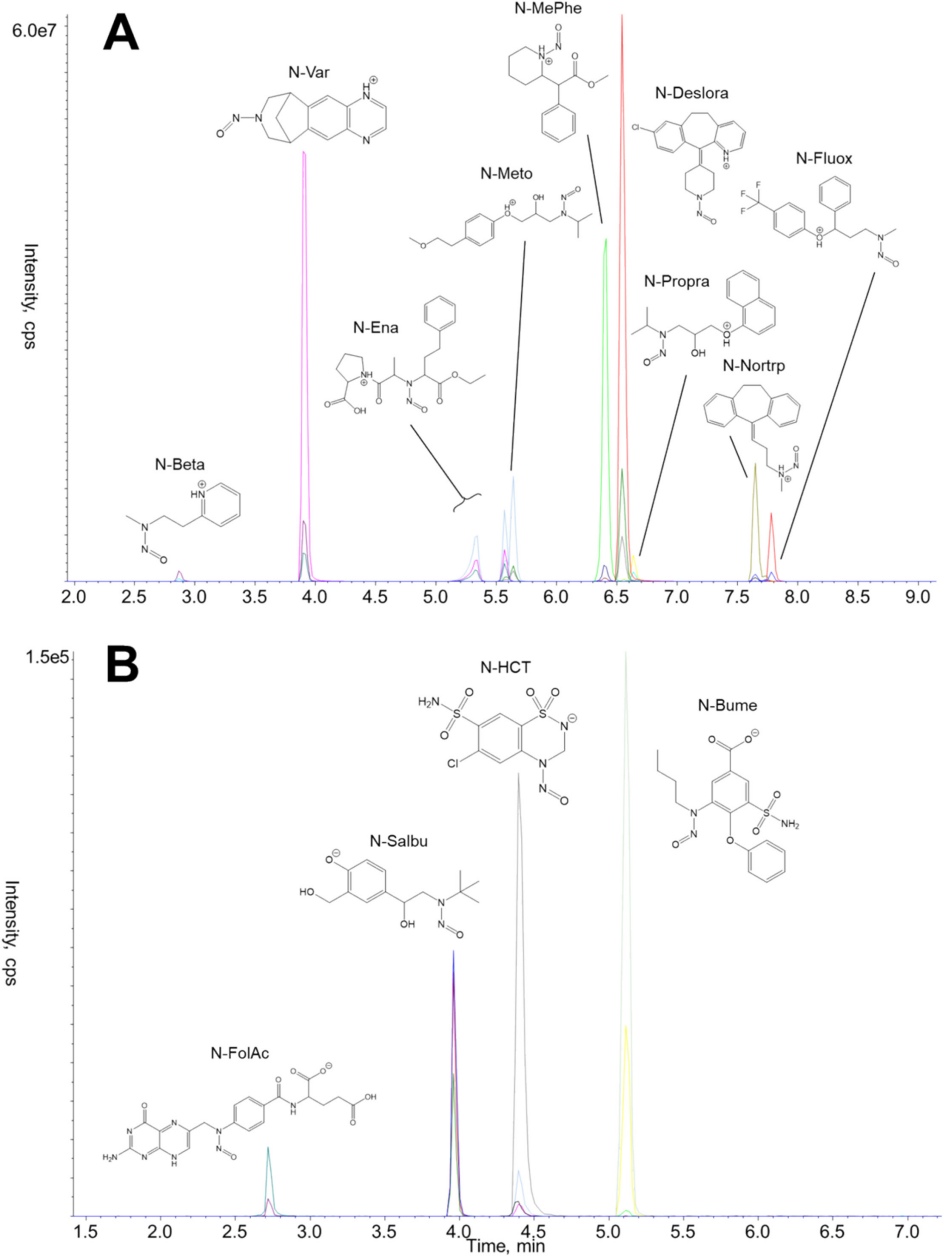
MRM chromatograms of an aqueous 5 nmol/mL NDSRI reference mixture determined under (A) positive ESI and (B) negative ESI conditions.

The combination of ammonium acetate buffer and acetonitrile provided adequate conditions to sufficiently ionize NDSRIs under positive and negative ESI conditions. Typical for nitrosamines was the occurrence of cis‐trans isomers (geometric isomers) often resulting in non‐resolved double peaks, which were observed for NProp, NNor, and NMeto. Additionally, NEna revealed two regions of isomers under the ascertained conditions, which can causally be related to further cis‐trans isomerism at the amide bond [20]. In contrast to the compounds metoprolol, propranolol, and nortriptyline, which could be clearly identified as single peaks in LC–MS chromatograms before they were converted into nitroso derivatives, enalapril already showed two signals here. The reason for this is the already present degree of more complex isomerism, which was further increased by the introduction of the nitroso group (data not shown). Due to a higher full width at half maximum (FWHM) value and unbeneficial fronting effects of the first peak, the second peaks at higher retention times of nitrosoenalapril and d5‐nitrosoenalapril were preferred for area integration and quantification. The insufficient stability of nitroso groups at DP > 80 [eV], in particular for compounds with adjacent mesomeric stabilized systems (e.g., nitrosamines from secondary aromatic amines), could be avoided by generally adapting the DP to 40 eV. On the other hand, losses of nitric oxide (NO, *m/z* 30) or nitroxyl (HNO, *m/z* 31) in the product ion spectra were pivotal to corroborate nitrosamine structure residues. For validation and quantification, the paired ion transitions of single protonated precursors and quantifiers were utilized. Exemplary positive as well negative ESI MRM chromatograms are depicted under Figure 2.

### 3.2 Validation

The method described and results of validation facilitate the implementation of analytical NDSRI methodology for routine screening purposes, but also to avoid compound‐related issues, which occurred during the measurements. All methods developed for analysis of NDSRIs, with exception of NHCT met all validation criteria of ICH Q2 (R1) and were therefore suitable for further quantitative investigations. Acceptance criteria were in concordance to ICH guideline M10 on bioanalytical method validation and study sample analysis. Linearity, precision and accuracy could not be fulfilled for NHCT within the acceptance criteria due to strong deviations in signal intensities. One reason that could explain the failure of the NHCT method to meet validation criteria is instability of NHCT under acidic conditions accompanied by slow degradation [21]. In contrast to stable NHCT stock solutions, instantly formed NHCT could quantitatively be detected in low concentrations within a period of approximately 2 h after acidic incubations. All investigated validation parameters are listed in Table 2. Results for the intermediate precision can be found in the supplementary information under Table S1.

**TABLE 2:** Determined results for validation for the investigated NDSRIs. Due to instability of nitrosohydrochlorothiazide, the parameters’ precision and accuracy were not performed. Acceptance criteria were *x* ≤ 15% relative SD for repeatability and accuracy and *x* > 0.99 for all coefficients of correlation.

Compound	LOD (pmol/mL)	LOQ (pmol/mL)	Repeatability (rel SD %)			Accuracy (rel SD %)			Linearity	
			0.025 nmol/mL	0.25 nmol/mL	2.5 nmol/mL	0.1 nmol/mL	1 nmol/mL	5 nmol/mL	Range (nmol/mL)	*R* ^2^
Nitrosobetahistine	1.25	5	8.0	6.0	15.0	109.3	102.2	96.5	0.005–5	0.997
Nitrosofolic acid	5	25	12.1	14.3	12.8	87.8	101.1	100.6	0.025–5	0.999
Nitrosodesloratadine	0.08	0.16	4.3	4.0	2.9	86.0	103.6	94.6	0.005–5	0.994
Nitrosoenalapril	0.16	0.32	13.6	5.5	9.1	95.4	106.3	98.3	0.005–5	0.999
Nitrosofluoxetine	0.32	0.64	13.8	14.2	7.6	90.8	89.7	97.2	0.005–5	0.995
Nitrosohydrochlorothiazide	50	250	—	—	—	—	—	—	0.25–5	0.969
Nitrosomethylphenidate	0.08	0.16	10.7	3.8	8.6	89.7	99.0	98.7	0.005–5	0.993
Nitrosometoprolol	0.08	0.16	8.3	10.0	14.2	89.4	99.4	90.6	0.005–5	0.994
Nitrosonortriptyline	1.25	2.5	6.1	11.1	11.8	114.8	113.9	108.6	0.005–5	0.993
Nitrosopropranolol	0.32	0.64	9.8	6.4	9.0	86.0	99.1	90.4	0.005–5	0.997
Nitrososalbutamol	2.5	5	10.3	11.5	13.8	90.6	103.7	101.6	0.005–5	0.991
Nitrosovarenicline	0.08	0.16	14.8	8.5	10.4	95.2	112.9	101.8	0.005–5	0.990
Nitrosobumetanide	0.16	0.64	12.3	8.0	12.5	91.0	102.4	94.7	0.005–5	0.991

#### 3.2.1 Specificity/Selectivity

For all 10 NDSRI free matrices, no interference or false positive signals were observed indicating reliability of the finally optimized work‐up procedure and LC–MS settings. Mass spectrometric selectivity and compound identifiability were confirmed by unambiguous ion transitions for each compound after NDSRIs and ISTD addition.

#### 3.2.2 Linearity/Range

Linearity was performed by means of linear regression of the area ratios between 0.005 and 5 nmol/mL. The working range was defined in accordance to the defined LOQ of each substance. Coefficients of correlation were set to *x* > 0.99 as acceptance criteria.

#### 3.2.3 LOD/LLOQ

Lower concentrations and departures from linearity ranges were tested for LOD and LOQ. For most compounds, signal‐to‐noise ratios of 3:1 and 10:1 could be reached even in the picomol per milliliter range, still fulfilling the LOD/LOQ requirements.

#### 3.2.4 Precision (Repeatability and Intermediate Precision)

With exception of NHCT, all replicates of the repeatability as well as threefold conducted repeatability/intermediate precision showed sufficient deviations below 15% as acceptance criteria for 0.025, 0.25, and 2.5 nmol/mL. Table 2 summarizes all values from repeatability parameter.

#### 3.2.5 Accuracy

Criteria of accuracy testing was applied to all NDSRIs, ensuring the quantitative result to be unaffected by systematic errors. For all NDSRIs, with partial or complete exception of NBeta, NFolAc, and NHCT, adequate accuracy values were determined within the acceptance criteria within the range of ±15%.

#### 3.2.6 Robustness

After column exchange, all NDSRI signals negligibly deviated from the prior measurements with regard to retention time. The order of compound elution also remained constant. Beneficially, signal intensities for some compounds showed an improvement in resolution.

#### 3.2.7 Stability

In the event of instability or degradation of substances, new derivatives of NDSRIs would be formed, which could possibly have impacts on further toxicity assays (e.g., AMES test, COMET assay). False positive or false negative results would be the consequence. It could be shown that the tests of NDSRIs have sufficient relative stabilities in DMSO and double distilled water at 4 and −20°C, respectively. This series of tests was performed for at least four consecutive days. Randomly selected samples were tested again after day 15, stored at the described conditions. Again, no substantial decrease in concentration could be detected, indicating sufficient stability in relevant solutions. All stabilities are listed graphically in the supplementary information (Figure S1).

### 3.3 Quantitative NDSRI Determinations Under Artificial Gastric Conditions

**FIGURE 3:**
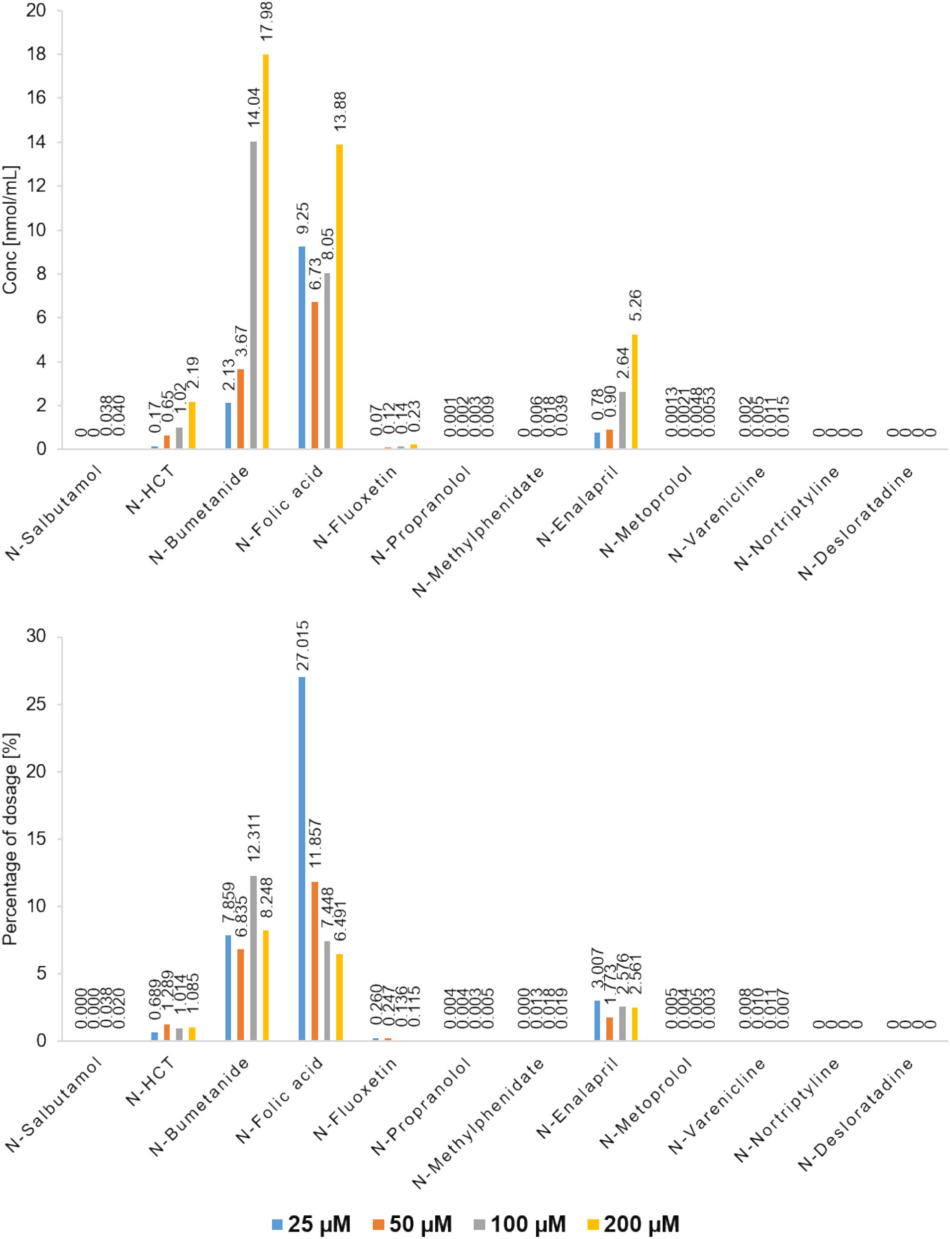
Quantification of NDSRIs formed under artificial gastric conditions with 25, 50, 100, and 200 μM of each API incubated with 200 μM nitrite at pH 3.15 and 37°C for 2 h. Results are depicted as concentration in nanomole per milliliter and percentage of the utilized API dosage. All technical replicates were performed with *n* = 2.

Prior to testing, a literature review was conducted to identify physiologically adequate concentrations for gastric nitrite, which can be expected under normal fasting conditions. Here, highly varying concentrations for gastric nitrite ranging from approximately 1 to 200 μM were identified and can significantly be affected by various impacts like food intake, health status, and microbiomal nitrate‐nitrite conversion in the saliva, which impeded the selection of adequate nitrite values [22, 23, 24]. To trigger the nitrosation rate, a physiologically reasonable higher nitrite concentration of 200 μM was therefore chosen, which reflects the upper limit of the reviewed literature. With regard to choosing the optimum pH for nitrosation, the assumption that if pk_a_ (sec amine) > 5, the maximum nitrosation rate is between pH 3 and 3.4 (near pk_a_ nitrous acid with 3.15) [25]. Therefore, the published optimum pH value for nitrosation of 3.15 was used for validation as well as for the artificial gastric incubations. The reason for the non‐conversion of betahistine to its nitroso derivative could not be clarified. It was evident that all secondary aromatic amines in form of bumetanide, folic acid, and hydrochlorothiazide reveal higher conversion rates in contrast to aliphatic counterparts. An explanation for this observation can be the low protonation at pH 3.15. These observations corroborate findings from Schlingemann et al., where amines with low pk_a_ are predicted to have higher nitrosation rates at low pH values compared with secondary amines with higher pk_a_. An additional experiment corroborate these findings where the here investigated APIs at 100 μM were incubated with four different sodium nitrite concentrations following an exponential growth to assess the different occurring rates of nitrosation. Afterwards, the obtained NDSRI areas were individually plotted against the nitrite concentration where the slope of linear regression analysis directly correlates with the grade of nitrosation per nitrite level. The semiquantitative depiction of the experiment as well as the linear regression plots can be found depicted under Figures S1 and S2 in the supplementary information. Interestingly, also enalapril, and its aliphatic secondary amine structure, showed higher nitrosation rates in contrast to other tested aliphatic amines. It should be mentioned that quantification was performed by expanding linearity to 50 nmol/mL deviating from the validated range with its limit at 5 nmol/mL. Nevertheless, even in this range, coefficients of correlation for linearities did not fall below 0.99. To note is the observation that both the prodrug loratadine and the tertiary amine amitriptyline did not show any conversion to corresponding NDSRIs in any of the tests, which indicates sufficient stability under the selected conditions. Both compounds were selected to investigate the nitrosation susceptibility of NDSRIs bearing tertiary amines (amitriptyline) or are used as prodrugs (loratadine). Here, nitrosating agents need to overcome high reaction energies to form positively charged iminium cation intermediates followed by subsequent dealkylations to secondary amines [7]. All results are summarized in Figure 3.

### 3.4 In Silico Analysis of Structure Activity Relationship

**FIGURE 4:**
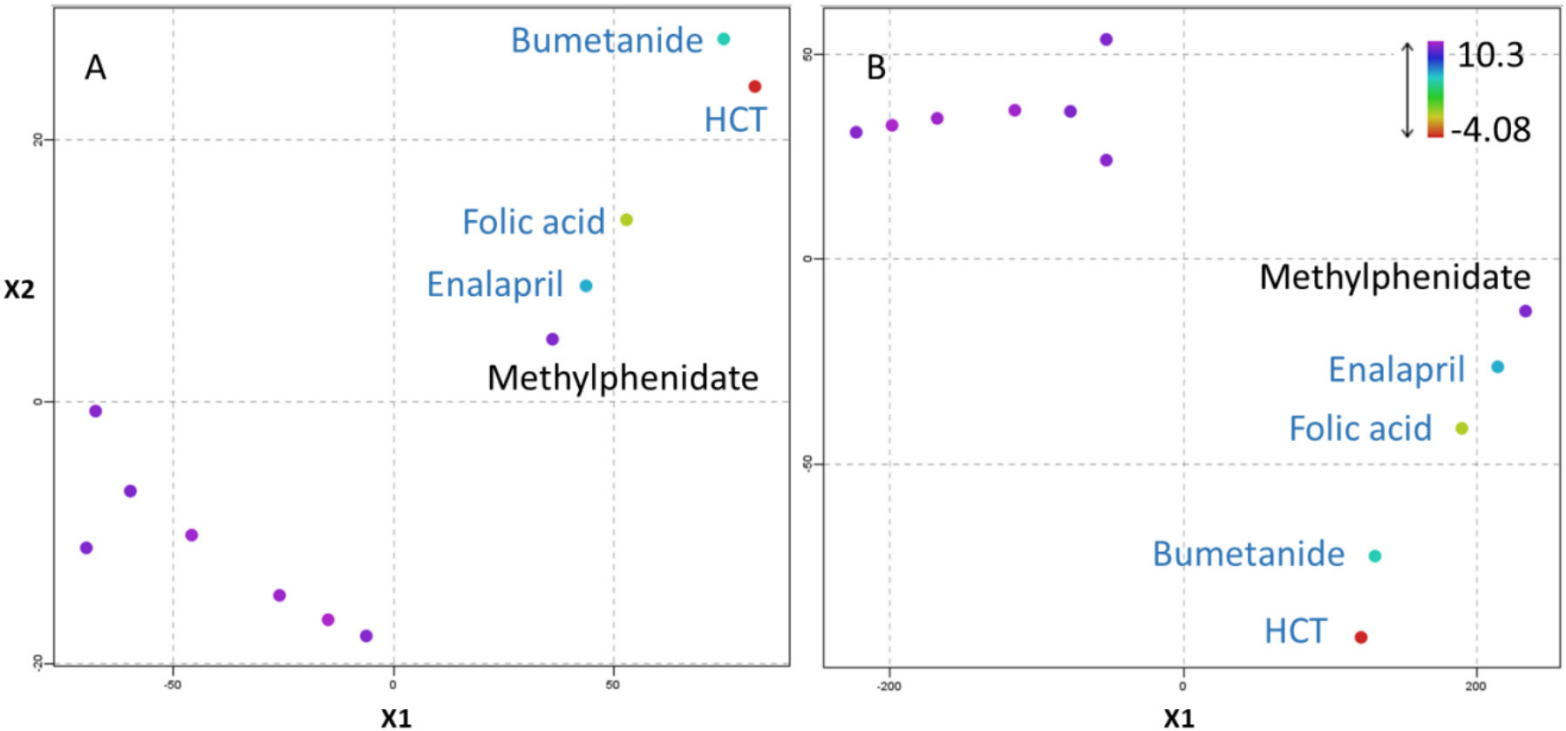
TSNE 2D scatterplot representation of the 12 APIs using the MACCS chemical fingerprint with the Tanimoto (A) and Dice (B) algorithm for similarity calculations. The color code represent the predicted pk_a_ values from Table 3.

Briefly, as outlined in the quantitative NDSRI determination by means of LC–MS, only four of the tested 12 APIs, namely, hydrochlorothiazide, enalapril, folic acid, and bumetanide, showed a conversion to their respective nitrosamine derivatives under acidic conditions within the exposure to different nitrite concentrations at pH 3 (Table 3). It is a challenge to derive robust structural rules that describe the ability of secondary amines to form nitrosamines under acidic conditions on the basis of this small data set. The feasibility of a read‐across approach was investigated based on the structural information of the 12 APIs. Exemplarily, Tanimoto or Dice scores were calculated for each combination of the APIs using the MACCS molecular fingerprint. The obtained scores were used to cluster the 12 APIs in the *t*‐distributed stochastic neighbor embedding (TSNE) plot to illustrate their chemical similarity (Figure 4). A TSNE plot is defined as an unsupervised nonlinear reduction technique and has been widely used to visualize similar chemical groups based on high dimensional data, for example, by Nelms and Patlewicz [26]. The four compounds susceptible to form NAs under acidic conditions (enalapril, folic acid, hydrochlorothiazide, and bumetanide) cluster nicely together, and no remarkable differences were observed between Tanimoto or Dice scores. Methylphenidate clusters relatively close to the four susceptible compounds and could be identified as positive if this clustering had been performed to search for compounds closely related to known susceptible compounds. One hypothesis is that protonated secondary amines are less susceptible to form NAs, as this reaction needs the electron lone pair of the amine to react with nitrite. Apparently, all APIs belonging to category 1, with the exception of enalapril, have relatively low pk_a_ values for the secondary amine and therefore are predicted to be not protonated at pH 3 (Table 3) (Figure 3). Enalapril, however, is predicted to have pk_a_ of 5.43 and therefore to be 100% protonated at pH 3. This study used in silico predicted pk_a_ values characterizing the ability of the secondary amine substructure to get protonated. This in silico model does not provide any information on its performance, for example, with regard to accuracy or applicability domain. Nonetheless, enalapril was converted to its NA derivative. The local structure around the secondary amine in enalapril is very different from the other APIs investigated in this project. It can be hypothesized that the two ketone groups exert an electron‐withdrawing effect that reduces the electron density at the secondary amines. This effect would reduce its nucleophilicity and thus its ability to get protonated. This hypothesis is better in line with the ability of enalapril to from the NA derivative.

**TABLE 3:** Selected pk_a_ values for the investigated APIs with protonation grades at pH 3. Cat_1 APIs do not form NAs, whereas Cat_2 APIs form NAs.

Name	CAS	SMILES	Sec amine (pk_a_)	Sec amine protonated (%) (ACD pred*); ph 3	Cat_1 (binary)
**Nortriptyline**	72–69–5	CNCCC=C1C2 = CC=CC=C2CCC3 = CC=CC=C31	10	100	0
**Desloratadine (see Loratadine)**	100,643–71–8	C1CC2 = C(C=CC(=C2)Cl)C(=C3CCNCC3)C4 = C1C=CC=N4	10.27	100	0
**Propranolol**	525–66–6	CC(C)NCC (COC1 = CC=CC2 = CC=CC=C21)O	9.5	100	0
**Metoprolol**	51,384–51–1	CC(C)NCC (COC1 = CC=C(C=C1)CCOC)O	9.43	100	0
**Varenicline**	249,296–44–4	C1C2CNCC1C3 = CC4 = NC=CN=C4C=C23	9.6	100	0
**Methylphenidate**	113–45–1	COC(=O)C(C1CCCCN1)C2 = CC=CC=C2	9.51	100	0
**Salbutamol**	18,559–94–9	CC(C)(C)NCC(C1 = CC(=C(C=C1)O)CO)O	9.62	100	0
**Fluoxetine**	54,910–89–3	CNCCC(C1 = CC=CC=C1)OC2 = CC=C(C=C2)C(F)(F)F	10.05	100	0
**Hydrochlorothiazide**	58–93–5	C1NC2 = CC(=C(C=C2S(=O)(=O)N1)S(=O)(=O)N)Cl	−4.08	0	1
**Enalapril**	75,847–73–3	CCOC(=O)C (CCC1 = CC=CC=C1)NC(C)C(=O)N2CCCC2C(=O)O	5.43	100	1
**Folic Acid**	59–30–3	C1 = CC(=CC=C1C(=O)NC (CCC(=O)O)C(=O)O)NCC2 = CN=C3C(=N2)C(=O)NC(=N3)N	−0.63	4	1
**Bumetanide**	28,395–03–1	CCCCNC1 = C(C(=CC(=C1)C(=O)O)S(=O)(=O)N)OC2 = CC=CC=C2	4.48	0	1

*Note:* The red color emphasizes the discrepancy between the relatively high conversion into nitrosoenalapril contrary to the unfavorable physicochemical parameters (protonation grade and pka value) for nitrosation.

Read‐across to known susceptible compounds (e.g., using structural parameters as shown here) as well as the knowledge on the protonation status of the secondary amine could be used as first screening tool to identify potentially susceptible APIs containing secondary amines.

## 4 Conclusion

The increased incidence of NDSRIs requires robust and safe methods to detect this type of contamination at an early stage to ensure drug quality and patient safety. It is not just the mere presence of nitrosamines that is significant here. Only the findings on the interplay between metabolic activation, stability and alkylation tendency of the released carbenium ions, and ultimately the cell's ability to repair the foreign molecule residue bound to the DNA, allow a correct toxicological assessment of the nitroso compound. This validated approach presented here constitutes a basis for the detection and characterization of further or similar NDSRIs. Thereby, common ESI conditions were applied for compound detection, facilitating the implementation of analysis with a variety of LC–MS devices. All NDSRIs tested so far could be qualitatively measured although there were challenges for the quantitative analysis of NHCT and partially for NFolAc in terms of linearity range, accuracy, and precision. Nevertheless, to individually evaluate endogenous nitrosation tendencies of selected APIs a quantitative approach under artificial gastric conditions was conducted. All APIs showed a conversion to nitrosamine derivatives. Of note, all secondary aromatic amines revealed higher tendencies of nitrosation in contrast to aliphatic forms, which was also confirmed by means of SAR. In conclusion, the majority of the investigated APIs form NAs in quantities in the lower nmol/mL range under acidic conditions. Whether these quantities are of toxicological significance cannot be assessed at this stage of the data situation. Here, more data from toxicological testings, for instance, AMES tests and/or COMET assays, are of utmost importance. All NA forming compounds have a pk_a_ value that indicate low to no protonation of the secondary amine at approximately pH 3. In contrast, enalapril is predicted to be 100% protonated at pH 3. Nonetheless, it forms the NA comparable with the other three category 1 compounds possibly due to electron‐withdrawing effect in the vicinity of the amine group.

## Disclosure

The statements presented does not convey the official positions of the BfArM or EMA.

## Conflicts of Interest

The authors declare no conflicts of interest.

## Supporting information


**Figure S1:** Semiquantitative determinations of NDSRI occurrence in dependency of nitrite concentrations. All APIs were incubated separately. Of particular interest was the change in the peak area when the nitrite concentration was increased by a factor of 10. The peak responses serve as a measure of the sensitivity to nitrosation. It can be seen that primary aromatic APIs in particular show a sometimes disproportionate response. Unexpectedly, enalapril, although an aliphatic secondary amine, also shows a significant increase. Measurements were conducted with *n* = 1.
**Figure S2:** Plotted change of areas per exponential increase of nitrite concentration. Thereby, the slope of each linear regression can be used amongst to assess the extent and susceptibility of nitrosation. The slope serves as a measure to evaluate and compare the susceptibility and propensity of the compounds examined to undergo nitrosation.
**Table S1: Intermediate Precision.** Taking repeatability into account, the intermediate precision was determined on three consecutive days and the relative standard deviations of the peak‐to‐area ratios between days 1 and 2, 1 and 3, and correspondingly 2 and 3 were calculated.

## Data Availability

The data that support the findings of this study are available from the corresponding author upon reasonable request.
