# Supplementary material for: Quantitative Investigation of Nitrosamine Drug Substance‐Related Impurities (NDSRIs) Under Artificial Gastric Conditions by Liquid Chromatography–Tandem Mass Spectrometry and Structure–Activity Relationship Analysis
Source: Drug Test Anal. 2025 Mar 12;17(9):1772–84. doi: 10.1002/dta.3874 (PMC12401632; doi:10.1002/dta.3874)
Supplement: Supplementary file 1 — Figure S1: Semiquantitative determinations of NDSRI occurrence in dependency of nitrite concentrations. All APIs were incubated separately. Of particular interest was the change in the peak area when the nitrite concentration was increased by a factor of 10. The peak responses serve as a measure of the sensitivity to nitrosation. It can be seen that primary aromatic APIs in particular show a sometimes disproportionate response. Unexpectedly, enalapril, although an aliphatic secondary amine, also shows a significant increase. Measurements were conducted with n = 1. Figure S2: Plotted change of areas per exponential increase of nitrite concentration. Thereby, the slope of each linear regression can be used amongst to assess the extent and susceptibility of nitrosation. The slope serves as a measure to evaluate and compare the susceptibility and propensity of the compounds examined to undergo nitrosation. Table S1: Intermediate Precision. Taking repeatability into account, the intermediate precision was determined on three consecutive days and the relative standard deviations of the peak‐to‐area ratios between days 1 and 2, 1 and 3, and correspondingly 2 and 3 were calculated. [file DTA-17-1772-s001.docx]

**Quantitative Investigation of Nitrosamine Drug Substance-Related Impurities (NDSRIs) under Artificial Gastric Conditions by Liquid Chromatography – Tandem Mass Spectrometry and Structure-Activity Relationship Analysis**

Matthias Vogel^1^, Sylvia E. Escher^2^, Emanuel Weiler^1^, Anke Londenberg^2^, Uwe Deppenmeier^3^, Rhys Whomsley^4^

^1^Federal Institute for Drugs and Medical Devices, Bonn, Germany

^2^Fraunhofer Institute for Toxicology and Experimental Medicine, Hannover, Germany
^3^Institute of Microbiology and Biotechnology, University of Bonn, Bonn, Germany

^4^European Medicines Agency, Amsterdam, The Netherlands

Corresponding author:

Dr. Matthias Vogel

Federal Institute for Drugs and Medical Devices

Kurt-Georg-Kiesinger-Allee 3

53175 Bonn

Germany

Tel: +49 228 99 307-3609

[matthias.vogel@bfarm.de](mailto:matthias.vogel@bfarm.de)

ORCID 0000-0003-4814-3900

**Keywords: Nitrosamines, NDSRIs, analysis, validation, liquid chromatography, mass spectrometry**

**Table S1: Intermediate Precision.** Taking repeatability into account, the intermediate precision was determined on three consecutive days and the relative standard deviations of the peak-to-area ratios between days 1 and 2, 1 and 3, and correspondingly 2 and 3 were calculated.

##### Results of the intermediate imprecision determined at three consecutive days in percent [%]

|  | **D1-D2** | | | **D1-D3** | | | **D2-D3** | | |
| --- | --- | --- | --- | --- | --- | --- | --- | --- | --- |
| **Compound** | 0.025 nmol/mL | 0.25 nmol/mL | 2.5 nmol/mL | 0.025 nmol/mL | 0.25 nmol/mL | 2.5 nmol/mL | 0.025 nmol/mL | 0.25 nmol/mL | 2.5 nmol/mL |
| Nitrosobetahistine | 14.5 | 6.5 | 11.3 | 15.0 | 14.2 | 14.9 | 9.5 | 14.2 | 12.9 |
| Nitrosofolic acid | 13.2 | 13.4 | 15.0 | 13.8 | 11.2 | 10.8 | 14.2 | 11.8 | 14.6 |
| Nitrosodesloratadine | 4.0 | 3.5 | 3.0 | 3.7 | 3.3 | 2.7 | 4.3 | 3.0 | 3.0 |
| Nitrosoenalapril | 11.7 | 6.3 | 7.2 | 11.0 | 6.2 | 6.2 | 5.2 | 5.2 | 4.4 |
| Nitrosofluoxetine | 10.2 | 13.6 | 6.5 | 11.3 | 13.4 | 12.6 | 12.9 | 0.9 | 12.9 |
| Nitrosohydrochlorothiazide | - | - | - | - | - | - | - | - | - |
| Nitrosomethylphenidate | 8.2 | 5.8 | 7.1 | 7.6 | 4.6 | 8.1 | 4.7 | 5.6 | 4.5 |
| Nitrosometoprolol | 8.0 | 10.2 | 9.8 | 7.7 | 8.0 | 12.6 | 7.4 | 9.3 | 8.6 |
| Nitrosonortriptyline | 13.5 | 14.6 | 12.9 | 10.4 | 11.5 | 8.1 | 13.7 | 12.3 | 12.6 |
| Nitrosopropranolol | 10.0 | 8.3 | 8.1 | 10.0 | 6.4 | 8.2 | 6.1 | 8.6 | 7.2 |
| Nitrososalbutamol | 11.9 | 10.7 | 13.2 | 11.9 | 14.5 | 10.6 | 13.4 | 14.9 | 11.4 |
| Nitrosovarenicline | 14.8 | 11.0 | 11.3 | 11.6 | 11.9 | 12.8 | 13.2 | 14.1 | 12.7 |
| Nitrosobumetanide | 12.2 | 9.9 | 9.2 | 11.4 | 7.6 | 12.1 | 12.0 | 7.9 | 12.9 |

**Figure S1:** Semiquantitative determinations of NDSRIs occurrence in dependency of nitrite concentrations. All APIs were incubated separately. Of particular interest was the change in the peak area when the nitrite concentration was increased by a factor of 10. The peak responses serve as a measure of the sensitivity to nitrosation. It can be seen that primary aromatic APIs in particular show a sometimes disproportionate response. Unexpectedly, enalapril, although an aliphatic secondary amine, also shows a significant increase. Measurements were conducted with n=1.


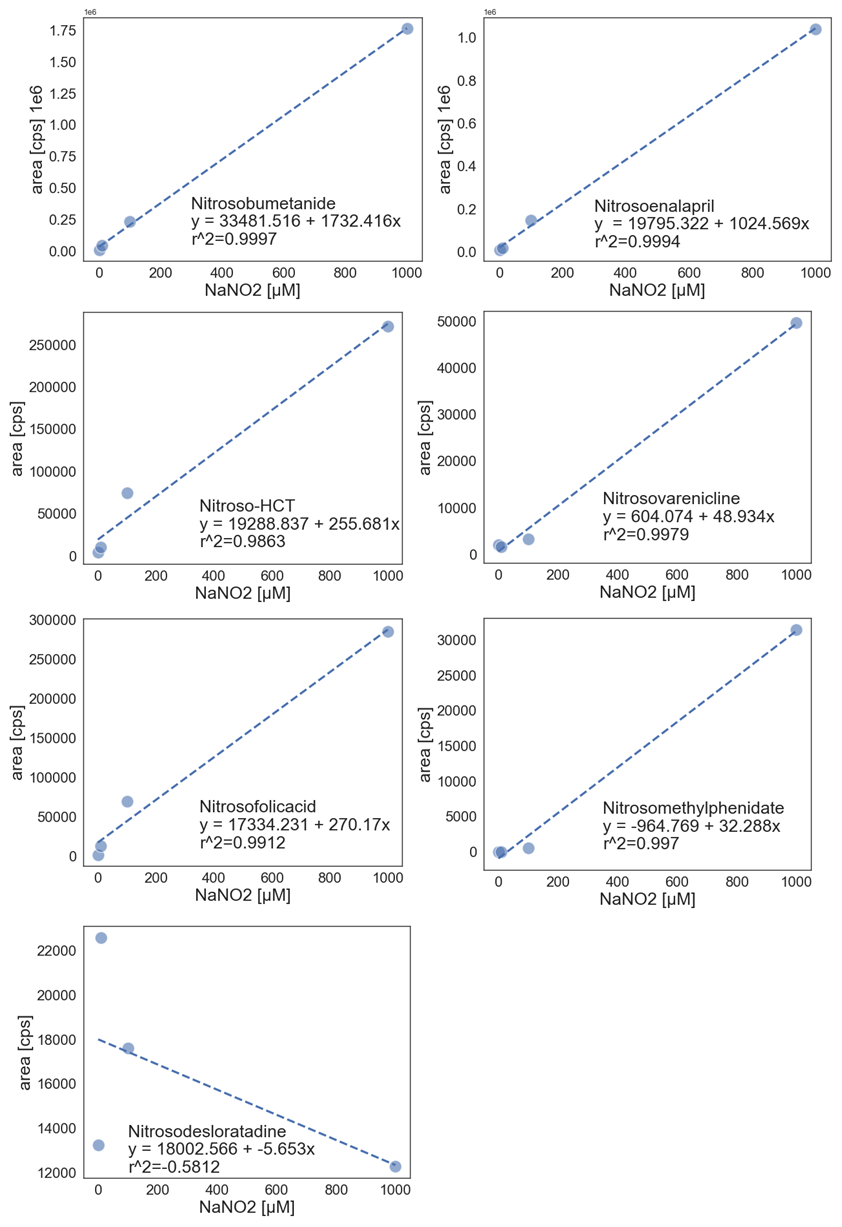


**Figure S2:** Plotted change of areas per exponential increase of nitrite concentration. Thereby, the slope of each linear regression can be used amongst to assess the extent and susceptibility of nitrosation. The slope serves as a measure to evaluate and compare the susceptibility and propensity of the compounds examined to undergo nitrosation.
